# The impact of diurnal sleep on the consolidation of a complex gross motor adaptation task

**DOI:** 10.1111/jsr.12207

**Published:** 2014-09-25

**Authors:** Kerstin Hoedlmoser, Juergen Birklbauer, Manuel Schabus, Patrick Eibenberger, Sandra Rigler, Erich Mueller

**Affiliations:** 1 Laboratory for Sleep, Cognition and Consciousness Research, Centre for Cognitive Neuroscience, University of SalzburgSalzburg, Austria; 2 Department of Sport Science and Kinesiology, University of SalzburgSalzburg, Austria

**Keywords:** gross motor learning, daytime sleep, REM, sleep spindles

## Abstract

Diurnal sleep effects on consolidation of a complex, ecological valid gross motor adaptation task were examined using a bicycle with an inverse steering device. We tested 24 male subjects aged between 20 and 29 years using a between-subjects design. Participants were trained to adapt to the inverse steering bicycle during 45 min. Performance was tested before (TEST1) and after (TEST2) training, as well as after a 2 h retention interval (TEST3). During retention, participants either slept or remained awake. To assess gross motor performance, subjects had to ride the inverse steering bicycle 3 × 30 m straight-line and 3 × 30 m through a slalom. Beyond riding time, we sophisticatedly measured performance accuracy (standard deviation of steering angle) in both conditions using a rotatory potentiometer. A significant decrease of accuracy during straight-line riding after nap and wakefulness was shown. Accuracy during slalom riding remained stable after wakefulness but was reduced after sleep. We found that the duration of rapid eye movement sleep as well as sleep spindle activity are negatively related with gross motor performance changes over sleep. Together these findings suggest that the consolidation of adaptation to a new steering device does not benefit from a 2 h midday nap. We speculate that in case of strongly overlearned motor patterns such as normal cycling, diurnal sleep spindles and rapid eye movement sleep might even help to protect everyday needed skills, and to rapidly forget newly acquired, interfering and irrelevant material.

## Introduction

According to a model by Fitts and Posner (1967), motor skill learning involves three stages: (i) cognitive stage (acquisition of explicit knowledge); (ii) associative stage (transfer from explicit to procedural knowledge); and (iii) autonomous stage (automatized performance). To adapt an automatized motor skill, the cognitive stage and then the associative stage have to be revisited. Regarding the time course of motor skill learning, a ‘fast learning phase’ (minutes; within-session improvement) and a ‘slow learning phase’ (further gains across several sessions of training) can be differentiated (Brashers-Krug *et al*., 1996; Doyon and Ungerleider, 2002; Karni *et al*., 1998). Furthermore, an intermediate phase occurring between practice sessions, during which the memory trace is believed to be processed offline, is thought to support motor memory consolidation (Doyon *et al*., 2009). The process of motor skill consolidation involves synaptic and systemic reorganizations of the neuronal representations underlying the motor skills, and finally leads to a robust and enduring memory trace (Dudai, 2004; McGaugh, 2000). However, a previously consolidated memory may become fragile and susceptible to interference again by recalling or reactivation (Nader, 2003; Stickgold and Walker, 2005). Therefore, a further period of re-consolidation might be required to modify, strengthen, change or even erase already consolidated memory.

It has been shown that after initial learning of a motor sequence task, skills are maintained over periods of wakefulness, and are in some cases even further enhanced following sleep (Korman *et al*., 2007; Walker *et al*., 2002). However, despite profound experience supporting the hypothesis that sleep plays a functional role in the consolidation of motor sequence learning (for a review, see Walker, 2005), the effect of sleep on the consolidation of motor adaptation tasks is still debated. On the one hand some papers (Doyon *et al*., 2009; Hill *et al*., 2008; Huber *et al*., 2004) reported sleep-related performance gains. On the other hand, savings in performance were consistently observed after retention interval irrespective of whether containing sleep or not, suggesting that the consolidation of motor adaptation tasks may not be dependent on sleep (Albouy *et al*., 2013a; Brashers-Krug *et al*., 1996; Debas *et al*., 2010; Donchin *et al*., 2002; Krakauer *et al*., 2005). Although the role of sleep in the offline processing of fine motor skills has been consistently investigated, data on complex gross motor learning are surprisingly rare (Blischke *et al*., 2008; Buchegger and Meier-Koll, 1988; Buchegger *et al*., 1991; Fogel and Smith, 2006; Kempler and Richmond, 2012; Morita *et al*., 2012), even if it is a very important memory domain essential for going through daily activities and the majority of tasks in competitive sports. In general, motor skills can be distinguished by the size of the muscle groups and by the number of biomechanical degrees of freedom required to perform different skills. Complex gross motor learning represents skills that use a large number of skeletal muscles and have several degrees of freedom, making it more difficult to master them (Magill, 2011; Wulf and Shea, 2002). In contrast to fine motor skills, gross motor skills are increasingly governed by gravitational and movement-dependent (inertial) forces (Schollhorn *et al*., 2009), providing a more complex pattern for movement control and, thus, at least initially, posing greater challenges to the cognitive capacity of the learner (Wulf and Shea, 2002). Therefore, there is an increasing consensus in motor science that theoretical and practical concepts on motor control and learning derived from the acquisition of fine motor skills cannot simply be transferred to complex gross motor tasks (Cordo and Gurfinkel, 2004; Schollhorn *et al*., 2009; Wulf and Shea, 2002). In an early study by Buchegger and Meier-Koll (1988), it has been demonstrated that subjects who were able to acquire new gross motor skills during multiple training units in trampolining over 8 weeks showed an increase in the duration of sleep-cycle, rapid eye movement (REM) and slow-wave sleep. Specifically, a correlation between motor learning and changes in sleep-cycle and REM duration was found. However, this increase of sleep-cycle duration as a consequence of trampolining exercise could not be replicated in a later study by Buchegger *et al*. (1991), whereas an increase in REM duration exclusively after trampolining was confirmed. Another study by Blischke *et al*. (2008) has examined the effects of sleep on a submaximal counter-movement jump, requiring subjects to produce a submaximal vertical force impulse of precisely 60% of the individual maximum. Investigating two groups who trained 12 h apart and returned for testing 12 h and 24 h following their training session, no differential effect of sleep and wakefulness could be revealed on performance on this gross motor task. In a more recent study by Kempler and Richmond (2012), participants were randomly assigned to either a sleep or a wake group, and were trained on an arm coordinated-reaching task. Gross motor skill performance improved in both groups following a night of sleep but not after a day of wakefulness. Whether 2 h diurnal sleep benefits complex motor skill learning was examined by Morita *et al*. (2012). Three-ball cascade juggling was improved after a 2h nap, whereas the control group staying awake did not show any improvement. In addition, increased slow oscillations as well as delta and sigma electroencephalogram (EEG) spectral power during non-REM (NREM) sleep after motor learning in comparison to a baseline nap without preceding learning was demonstrated.

The aim of our current study was to investigate the effects of a midday nap (2 h) on complex gross motor consolidation by means of learning to adapt cycling to an inverse steering device (see supplemental Video S1). The utilized bicycle is a self-built, common upright, two-pedal cycle with a fixed gear ratio. The steering is constructed by two equal gear wheels such that the bicycle has to be inversely controlled by mirrored steering movements. Beyond riding curves, as is the case in normal cycling, compensatory movements are needed to hold balance at speeds up to 6–8 m/s (Wilson *et al*., 2004). Because lower extremities have to be additionally controlled to produce appropriate propulsion, more than 50% of the skeletal muscles are involved in the motor task (Seiler, 1995), thus constituting a highly complex, whole-body gross motor skill. That kind of gross motor task comprises various interesting new and innovative aspects: (i) ecological validity, as it is a common demand in everyday life to ‘re-learn’ an already existing movement pattern or to adapt an highly automated movement pattern to a new situation; (ii) complexity and gross motor property by means of many degrees of freedom and the great number of muscles involved; (iii) the use of mainly implicit adaptation strategies under high time pressure that are required to hold balance; and (iv) based on this new task features, we expected to gain information on the general validity or task-dependency of sleep-related motor memory consolidation. As a consequence, we aimed at shedding light on the rather controversial picture presented throughout the sleep and motor memory consolidation literature that might be attributed to the fact that the characteristics of the investigated tasks largely differ between each other.

## Materials and Methods

Twenty-four healthy male subjects aged between 20 and 29 years (M = 24.08 years, SD = 2.12) were recruited from a student population. Exclusion criteria were as follows: history of drug/alcohol abuse; subjects who work at night; habitual nappers; a transatlantic trip 3 months prior to the study entrance, or any circadian phase-shifting condition; a Pittsburgh Sleep Quality Index global score above normal range (>5); unusual bedtimes and/or extreme chronotype; anxiety or depression. Participants engaged in professional bicycling regularly (more than once per week for a period of several hours) as well as high-level sportsmen in any other discipline were excluded from the study. For participation students received ECTS (European Credit Transfer System) points. All subjects were informed in detail about the project and gave their written informed consent before study inclusion. The study was performed in accordance with the national legislation for the protection of human volunteers in non-clinical research settings and the Declaration of Helsinki.

### Experimental design

**Figure 1:**
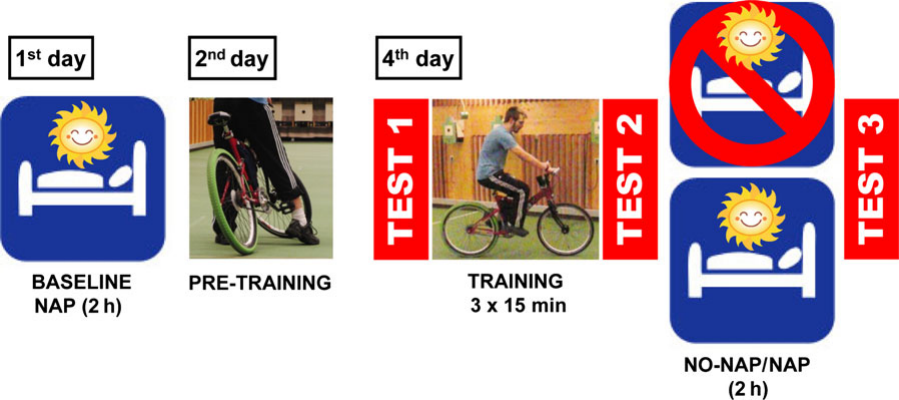
Study design. After taking a baseline nap on the first day, participants had to learn to handle (i.e. riding the bicycle three times for 30 m without dismounting) the inverse steering bicycle within a pre-training phase on the second day. On the fourth day, each subject performed an initial test (TEST1) including three times 30 m straight-line and three times 30 m slalom riding before participating in the 45-min exploratory training session, which was followed by a second test session (TEST2). Afterwards, subjects in the NAP group took a 2 h nap from 13:00 to 15:00 hours, while subjects of the NO-NAP group stayed awake for 2 h watching a BBC documentary. In both conditions, participants were tested for gross motor performance subsequent to the NAP/NO-NAP retention interval (TEST3).

All participants underwent an entrance examination including a clinical evaluation of sleep quality, mood disorders and chronotype. Throughout the whole examination period subjects had to wear wrist actigraphy (Cambridge Neurotechnologies, Cambridge, UK) and to complete a sleep diary every evening and morning. As depicted in Fig. 1, subjects came to the sleep laboratory on the first day for a polysomnography (PSG)-monitored baseline nap in the afternoon (13:00–15:00 hours). We used a 2 h nap protocol to increase the likelihood of subjects to reach both NREM and REM sleep. This baseline nap served adaptation purposes to make sure that subjects were able to nap during the day without (partial) sleep deprivation. Note that none of the subjects had to be excluded due to low sleep quality. On day 2, subjects came to a pre-training session (cf. Fig. 1, ‘PRE-TRAINING’) where they were instructed to exploratory learn to handle the inverse steering bicycle by self-paced trial and error. The pre-training required subjects to reach a specific criterion within 2 h: riding the inverse steering bicycle for three runs of 30 m without dismounting. This criterion was reached in 88 min on average (SD = 55 min). However, four out of the 24 subjects had to be excluded from the study as they did not reach the criterion. The aim of this pre-training was to familiarize the subjects with the gross motor task, provide similar pre-conditions for all participants and that the task could be performed at all. Performance was not recorded during this pre-training session. On day 4, subjects were required to perform an additional 45-min training session. Preceding this training session, performance of inverse cycling was assessed (after cycling warm-up) by three runs of 30 m: (i) straight-line; and (ii) slalom riding (TEST1). For (i), subjects were instructed to ride as straight as possible in parallel to lines that were marked on the floor in intervals of 50 cm. For (ii), they had to perform a slalom course (six pole bases equally spaced with 50 cm off-centre) by steering as little as possible along the sinusoidal path. Importantly, both tests were constructed to evaluate the riding performance to a different degree of difficulty rather than to test *per se* two different riding skills. A rotatory potentiometer mounted in the head tube of the inverse cycle was used to estimate the test performance by the standard deviation of the steering angle (SDSA) recorded throughout straight and slalom rides. In addition to steering accuracy, riding time was measured. As for the pre-training session, performance was not recorded during training and the nature of training was self-instructed and exploratory. During the training session, subjects were encouraged to practice and prepare as well as possible for straight-line and slalom riding. The training was followed by a second test (TEST2). After TEST2, each subject was randomly assigned either to group (A) NAP or (B) NO-NAP. Time-of-day confounds were controlled by testing nappers and non-nappers at the same time of day. Whereas subjects of the NAP group took a nap from 13:00 to 15:00 hours, the NO-NAP group remained awake while watching a BBC documentary (Downer *et al*., 2006). To ensure that subjects of the NO-NAP group did not fall asleep, PSG recordings and visual monitoring were used. After this 2 h retention interval, gross motor performance was tested again (TEST3). The retest was conducted approximately 15–30 min (M = 18 min, SD = 5 min) after the retention interval. All subjects completed questionnaires for sleepiness, affectivity, arousal, mood, drive and participation [100-mm visual analogue scales (ASES); Folstein and Luria, 1973; Stanford Sleepiness Scale (SSS); MacLean *et al*., 1992; ‘Mehrdimensionaler Befindlichkeitsfragebogen’ (MDBF); Steyer *et al*., 1997] before (i.e. after TEST2) and after (i.e. before TEST3) the retention interval (see supplemental Table S1).

### Polysomnography

During the baseline nap and the nap after training, PSG was recorded using an ambulatory 16-channel amplifier (Varioport©, Becker Meditec, Karlsruhe, Germany). PSG started at 13:00 hours and was terminated after 2 h time in bed irrespective of total sleep time. Data were recorded referentially against a common reference at Cz and off-line re-referenced to contralateral mastoids. PSG recordings including 12 EEG channels (F3, Fz, F4, C3, C4, P3, Pz, P4, O1, O2, A1, A2), two horizontal electrooculogram (EOG) channels, two vertical EOG and two submental electromyogram channels were obtained at a sampling rate of 512 Hz. Sleep was pre-scored and pre-staged automatically (Somnolyzer 24 × 7; Koninklijke Philips N.V., Eindhoven, The Netherlands) according to AASM criteria (Iber *et al*., 2007). Scoring and staging were controlled by visual inspection of an expert scorer. Sleep spindles during sleep stage N2 were detected automatically (ASK analyser; The Siesta Group, Vienna, Austria) using central (C3) electrodes, re-referenced to contralateral mastoids. Spindle detection was based on the following criteria: (i) 11–15 Hz band-pass filtering; (ii) amplitude >25 μV; (iii) duration > 0.5 s; and (iv) controlling for muscle (30–40 Hz) and/or alpha (8–12 Hz) artefacts (Anderer *et al*., 2005). Instead of the mean number of sleep spindles per 30 s (i.e. spindle density), spindle activity (SpA) was estimated using a measure that captures the duration as well as the amplitude of identified spindles (i.e. SpA = mean spindle duration * mean spindle amplitude).

### Statistical analyses

Statistical analyses were performed using PASW Statistics 18.0.2 software (SPSS, Chicago, IL, USA). Shapiro–Wilk tests were applied to test for the normality of the distribution of the data, which was given in all cases. The significance level was set to *P* < 0.05. Effect sizes are provided as eta squared (*η*^2^). For the training session on day 4, changes in gross motor performance, by means of straight-line and slalom riding [SD of steering angle (°) and riding time (s)], were evaluated by two-factor analyses of variances (anova) for repeated measures with the within-subject factor TEST (TEST1 versus TEST2) and the between-subjects factor GROUP (nap versus no-nap). Note that due to technical problems, we are only able to report nine values of SD of steering angle during the slalom ride (one missing) for TEST1. To investigate the impact of a retention interval either containing sleep or wakefulness on gross motor performance, we further conducted two-factor anovas with the within-subject factor TEST (TEST2 versus TEST3) and the between-subjects factor GROUP (nap versus no-nap). To observe differences between the two groups in fatigue (ASES, MDBF and SSS), we applied independent-samples *t*-tests. In order to control for sleep inertia after the retention interval, we additionally calculated analyses of covariance (ancova). Therefore, ASES, MDBF and SSS (with their inter-correlation being *r* > 0.76) were reduced to a common factor ‘fatigue’ by principal component analysis and used as a covariate examining the effects of TEST (TEST2 versus TEST3) and GROUP (nap versus no-nap). Differences in sleep parameters between baseline and experimental nap were evaluated by paired-samples *t*-tests. Pearson correlations (two-tailed) were used to test for linear relationships between over-nap changes in gross motor performance and SpA as well as REM duration during experimental NAP.

## Results

### Gross motor performance

Gross motor performance before (TEST1) and after training (TEST2) as well as after retention interval (TEST3) are shown in Table 1.

**Table 1:** Gross motor performance (riding time [s], standard deviation of steering angle – SDSA [°]) before training (TEST 1), after training (TEST 2) and after retention interval (TEST 3) differentiating between the two groups (NAP versus NO-NAP)

	TEST 1			TEST 2			TEST 3			Paired-samples *t* test			
										TEST 1 – TEST 2		TEST 2 – TEST 3	
	NAP mean ± SD	NO-NAP mean ± SD	*t* tests	NAP mean ± SD	NO-NAP mean ± SD	*t* tests	NAP mean ± SD	NO-NAP mean ± SD	*t* tests	NAP	NO-NAP	NAP	NO-NAP
STRAIGHT – LINE													
SDSA [°]	15.34 ± 4.56	13.47 ± 2.60	*t* = 1.125	8.75 ± 2.22	9.21 ± 2.88	*t* = −0.393	10.80 ± 2.87	10.23 ± 3.31	*t* = 0.408	*t* = 6.004	*t* = 8.216	*t* = −3.506	*t* = −2.587
			*P* = 0.279			*P* = 0.699			*P* = 0.688	***P*** **< 0.001**	***P*** **< 0.001**	***P*** **= 0.007**	***P*** **= 0.029**
Riding time [s]	17.23 ± 3.76	16.33 ± 3.33	*t* = 0.569	14.28 ± 2.71	14.40 ± 3.22	*t* = −0.092	15.85 ± 2.71	15.23 ± 3.86	*t* = 0.413	*t* = 2.158	*t* = 1.495	*t* = −1.854	*t* = −0.915
			*P* = 0.576			*P* = 0.927			*P* = 0.685	*P* = 0.059	*P* = 0.169	*P* = 0.097	*P* = 0.384
SLALOM													
SDSA [°]	21.88 ± 3.56	20.66 ± 2.34	*t* = 0.870	18.25 ± 1.30	18.76 ± 1.68	*t* = −0.770	20.35 ± 2.68	17.90 ± 1.55	*t* = 2.497	*t* = 2.761	*t* = 2.148	*t* = −2.637	*t* = 1.778
			*P* = 0.399			*P* = 0.452			***P*** **= 0.025**	***P*** **= 0.025**	*P* = 0.060	***P*** **= 0.027**	*P* = 0.109
Riding time [s]	25.47 ± 7.43	26.82 ± 6.60	*t* = −0.429	19.19 ± 3.24	20.04 ± 5.51	*t* = −0.419	20.75 ± 4.59	20.02 ± 5.41	*t* = 0.325	*t* = 3.894	*t* = 6.135	*t* = −1.876	*t* = 0.024
			*P* = 0.673			*P* = 0.682			*P* = 0.749	***P*** **= 0.004**	***P*** **< 0.001**	*P* = 0.093	*P* = 0.982

Data are presented as mean ± SD. Independent-samples *t* tests (dark grey) depict the differences in performance between NAP and NO-NAP for all three testing times (TEST 1, TEST 2, TEST 3); paired-samples *t* tests (light grey) show changes in performance over training (TEST 1 – TEST 2) and over retention interval (TEST 2 – TEST 3), respectively; *P*-values printed in bold represent statistical significant differences (*P* < 0.05); statistical trends (*P* < 0.10) are underlined.

### Impact of training on straight-line and slalom riding

**Figure 2:**
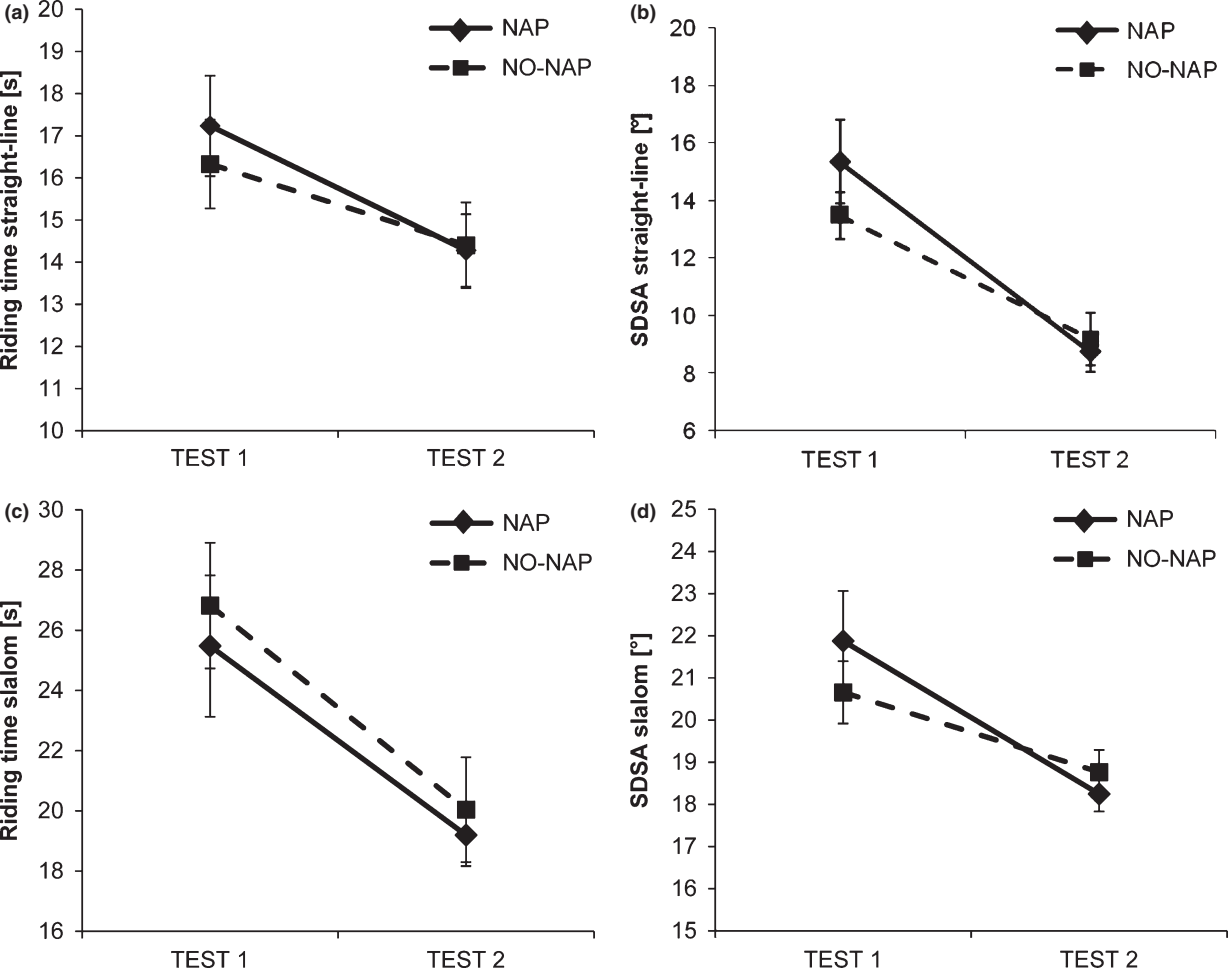
Impact of training on gross motor performance. Riding time (s) and steering accuracy (SDSA; standard deviation of steering angle) were decreased after training during straight-line (a, b) and slalom riding (c, d). Note: high SDSA values indicate low steering accuracy. Error bars represent standard error of mean.

Riding time (TEST: *F*_1,18_ = 6.738, *P* = 0.018, *η*^2^ = 0.269; cf. Fig. 2a) and accuracy (TEST: *F*_1,18_ = 79.975, *P* < 0.001, *η*^2^ = 0.787; cf. Fig. 2b) during straight-line riding were significantly enhanced after training. In contrast there were no significant group (riding time: *F*_1,18_ = 0.121, *P* = 0.732, *η*^2^ = 0.007; accuracy: *F*_1,18_ = 0.300, *P* = 0.591, *η*^2^ = 0.016) or interaction (riding time: *F*_1,18_ = 0.299, *P* = 0.591, *η*^2^ = 0.012; accuracy: *F*_1,18_ = 3.651, *P* = 0.072, *η*^2^ = 0.036) effects, showing that subjects of both groups similarly improved on straight-line riding due to the training. The same effects could be revealed for slalom riding: riding time (TEST: *F*_1,18_ = 44.620, *P* < 0.001, *η*^2^ = 0.712; cf. Fig. 2c) and accuracy (TEST: *F*_1,17_ = 12.628, *P* = 0.002, *η*^2^ = 0.410; cf. Fig. 2d) were improved after training. Again, there were no significant group (riding time: *F*_1,18_ = 0.200, *P* = 0.660, *η*^2^ = 0.011; accuracy: *F*_1,17_ = 0.249, *P* = 0.624, *η*^2^ = 0.014) or interaction (riding time: *F*_1,18_ = 0.066, *P* = 0.801, *η*^2^ = 0.001; accuracy: *F*_1,17_ = 1.177, *P* = 0.293, *η*^2^ = 0.038) effects, justifying that subjects of both groups have similarly gained their slalom riding performance with the inverse steering bicycle due to training.

### Impact of sleep versus wakefulness on straight-line and slalom riding

**Figure 3:**
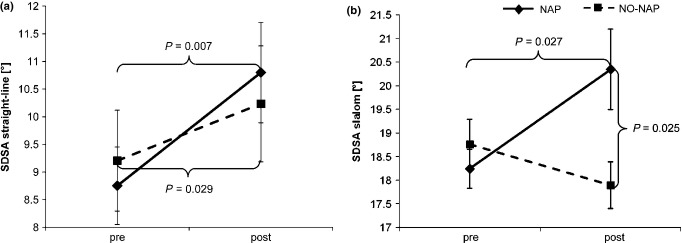
Impact of sleep versus wakefulness on gross motor performance. (a) Steering accuracy during straight-line riding (SDSA; standard deviation of steering angle) was decreased after sleep and wakefulness. (b) SDSA during slalom riding was decreased after nap but not after no-nap. Note: high SDSA values indicate low steering accuracy. Error bars represent standard error of mean.

Both groups showed a reduced straight-line riding performance after the retention interval as riding accuracy significantly decreased (TEST: *F*_1,18_ = 18.958, *P* < 0.001, *η*^2^ = 0.486; cf. Fig. 3a). Also for riding time we found a marginally significant main effect for TEST (*F*_1,18_ = 3.735, *P* = 0.069, *η*^2^ = 0.169). No significant group (riding time: *F*_1,18_ = 0.038, *P* = 0.848, *η*^2^ = 0.002; accuracy: *F*_1,18_ = 0.002, *P* = 0.963, *η*^2^<0.001) or interaction (riding time: *F*_1,18_ = 0.351, *P* = 0.561, *η*^2^ = 0.016; accuracy: *F*_1,18_ = 2.083, *P* = 0.166, *η*^2^ = 0.053) effects were found for both performance parameters in the straight-line riding condition. In the slalom test, no significant main (TEST: *F*_1,18_ = 1.840, *P* = 0.192, *η*^2^ = 0.085; GROUP: *F*_1,18_ = 0.001, *P* = 0.977, *η*^2^<0.001) or interaction (*F*_1,18_ = 1.929, *P* = 0.182, *η*^2^ = 0.089) effects for riding time were observed, while riding accuracy was found to significantly interact between TEST and GROUP (*F*_1,18_ = 10.093, *P* = 0.005, *η*^2^ = 0.338; cf. Fig. 3b). *Post hoc* tests revealed that there was a significant decrease in slalom performance after nap (*P* = 0.027), whereas after wakefulness performance remained stable (*P* = 0.109); slalom performance was significantly lower after nap in comparison to wakefulness (*P* = 0.025). Main effects for riding accuracy during slalom were not significant (TEST: *F*_1,18_ = 1.763, *P* = 0.201, *η*^2^ = 0.059; GROUP: *F*_1,18_ = 1.912, *P* = 0.184, *η*^2^ = 0.096).

### Effects of sleep inertia

Fatigue scores (MDBF, SSS and ASES) are listed in Table 2. Independent-samples *t*-tests revealed that subjects who took a nap during the retention interval tended to be less tired (MDBF: *t*_18_ = 1.782, *P* = 0.092, *η*^2^ = 0.15; SSS: *t*_18_ = −2.374, *P* = 0.029, *η*^2^ = 0.24; ASES: *t*_18_ = −1.657, *P* = 0.115, *η*^2^ = 0.13) after the retention interval in comparison to those subjects who stayed awake.

**Table 2:** Sleepiness scores (MDBF, SSS, ASES) before and after retention interval

	PRE RETENTION INTERVAL				POST RETENTION INTERVAL			
	NAP (*n* = 10) mean ± SD	NO-NAP (*n* = 10) mean ± SD	*t*(18)	*P*	NAP (*n* = 10) mean ± SD	NO-NAP (*n* = 10) mean ± SD	*t*(18)	*P*
Sleepiness								
MDBF	14.20 ± 2.74	12.10 ± 3.41	1.517	0.147	13.20 ± 4.39	9.90 ± 3.87	1.782	0.092
SSS	2.40 ± 0.97	2.78 ± 1.30	−0.723	0.479	2.70 ± 1.16	3.90 ± 1.10	−2.374	**0.029**
ASES	40.20 ± 22.82	41.67 ± 30.04	−0.121	0.905	37.20 ± 29.60	57.80 ± 25.86	−1.657	0.115

Data are presented as mean ± SD. *t*- and *P*-values indicate statistical differences between the two groups (nap versus no-nap). Note: High scores at the ASES and the SSS indicate sleepiness, whereas the opposite is true for the MDBF where lower scores represent higher fatigue; *P*-values printed in bold represent statistical significant differences (*P* < 0.05); statistical trends (*P* < 0.10) are underlined.

Additionally, ancovas examining the effects of the combined covariate ‘fatigue’ (including MDBF, SSS and ASES) revealed that changes in straight-line riding time after sleep and wakefulness were not significantly influenced by the covariate ‘fatigue’ (*F*_1,17_ = 3.515, *P* = 0.078, *η*^2^ = 0.132). Note that the statistical trend for a main effect for the factor TEST was not affected (*F*_1,17_ = 4.257, *P* = 0.055, *η*^2^ = 0.159). Additionally, changes in accuracy of straight-line riding after the retention interval did not interact with the covariate ‘fatigue’ (*F*_1,17_ = 0.099, *P* = 0.757, *η*^2^ = 0.003) and the main effect for the factor TEST (*F*_1,17_ = 18.009, *P* = 0.001, *η*^2^ = 0.495) remained significant. Regarding riding accuracy during slalom, the ancova revealed a significant interaction with fatigue (*F*_1,17_ = 7.173, *P* = 0.016, *η*^2^ = 0.154). However, the TEST × GROUP interaction for SD of steering angle during slalom became even more pronounced (*F*_1,17_ = 20.113, *P* < 0.001, *η*^2^ = 0.431). Taken together, these results suggest that despite the fact that subjects were less tired in the nap group, they showed worse performance at retest.

### Sleep parameters

Sleep parameters for baseline and experimental nap are illustrated in Table 3. Paired-samples *t*-tests revealed that subjects spent longer times in stage N2 sleep (*t*_9_ = −2.405, *P* = 0.040, *η*^2^ = 0.39) during experimental nap.

**Table 3:** Descriptive statistics of sleep parameters during baseline and experimental nap

	Baseline mean ± SD	Experimental mean ± SD	t_(*9*)_	*P*
TIB (min)	119.70 ± 2.58	121.10 ± 1.24	−1.353	0.209
TST (min)	70.40 ± 29.73	73.50 ± 34.49	−0.303	0.769
SEFF (%)	59.16 ± 25.51	60.50 ± 28.06	−0.163	0.874
WASO (min)	30.15 ± 29.63	34.55 ± 28.67	−0.473	0.647
SOL (min)	19.40 ± 10.24	13.20 ± 9.07	1.447	0.182
N1 (%)	32.05 ± 26.90	25.89 ± 19.58	0.805	0.442
N2 (%)	**40.27 ± 14.04**	**52.27 ± 17.23**	−**2.405**	**0.040**
N3 (%)	7.50 **±** 12.46	6.40 **±** 11.69	0.240	0.816
REM (%)	20.18 ± 19.32	15.44 ± 14.27	0.757	0.468
SpA	16.00 ± 2.82	15.45 ± 3.02	0.930	0.377

Data are presented as mean ± SD. Paired-samples *t*-tests show statistical differences between the two naps (baseline versus experimental). Note: time in bed (TIB), total sleep time (TST), wake after sleep onset (WASO) and latency to the first period of N2 (SOL) in minutes; sleep efficiency (SEFF), time spent in stage N1, stage N2, stage N3 and rapid eye movement (REM) as a percentage of total sleep time; N2 sleep spindle activity (SpA, 12–15 Hz, C3).

*P*-values printed in bold represent statistical significant differences (*P* < 0.05).

**Figure 4:**
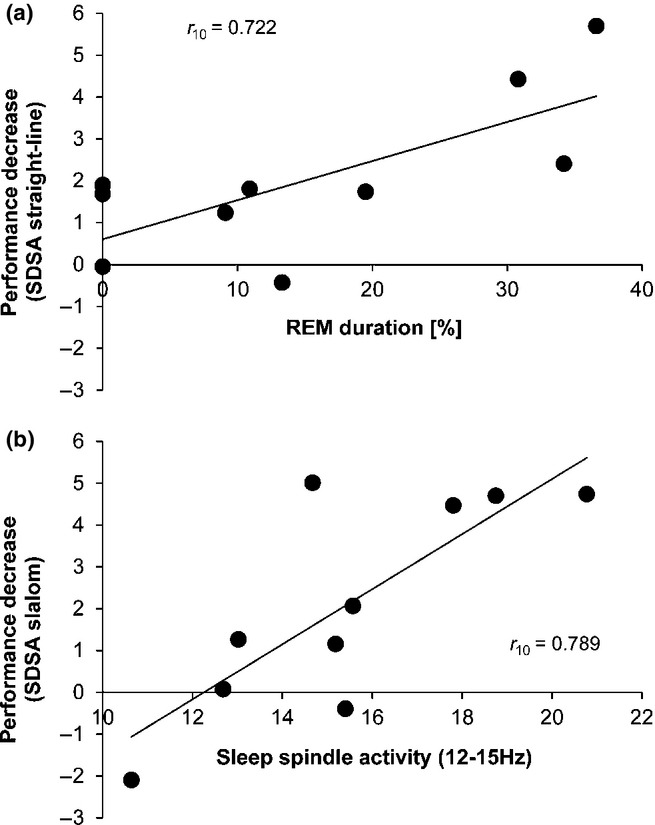
Sleep parameters and over-nap performance decrease. (a) REM duration (%) was negatively related to the change of straight-line riding performance over nap (standard deviation of steering angle, SDSA). (b) N2 sleep SpA (12–15 Hz, C3) was negatively related to the change of slalom riding performance over nap (SDSA). Note: high SDSA values indicate low steering accuracy.

We found a significant negative correlation between time spent in REM sleep during experimental nap and the oversleep change in straight-line riding (*r*_10_ = 0.855, *P* = 0.002; cf. Fig. 4a). The more time subjects spent in REM the worse was straight-line riding after nap. Furthermore, sleep SpA during experimental nap was strongly negative related to the oversleep change in slalom riding (*r*_10_ = 0.789, *P* = 0.007; cf. Fig. 4b). Subjects with high sleep SpA during nap after gross motor learning showed higher SDSA, indicating a more unstable handling of the bicycle.

## Discussion

In this study, subjects were trained to adapt to a new bicycle steering device. Effects on gross motor consolidation of diurnal sleep in comparison to wakefulness after training were investigated. We found that due to the training, all subjects were able to improve their riding time and accuracy during straight-line riding and slalom riding with the new steering device. However, we assume that participants definitely did not reach asymptotic performance at the end of the training. As in other complex gross motor tasks (e.g. bike trialing, playing golf), no ceiling effects were expected after this relatively short period of training as those kind of skills can be improved even after years of experience because of the vast amount of degrees of freedom to be controlled with high accuracy requirements. According to earlier findings (Hauptmann *et al*., 2005; Karni and Sagi, 1993; Korman *et al*., 2007), the fact that subjects did not reach a plateau of performance might be a problem for generating delayed gains in performance. However, note that those earlier studies focused on visual skills (visual discrimination task, enumeration task) or motor sequence learning (finger-to-thumb opposition task), further implying that motor memory consolidation seems to differ depending on the nature of the task.

Our results indicate a robust negative impact of a 2 h diurnal retention interval on the gross motor task. Both riding time and accuracy in straight-line riding were reduced after a 2 h retention interval independently whether retention contained sleep or wakefulness. Accuracy in the slalom-course was decreased after a 2 h nap, but remained stable over 2 h restful wakefulness. These behavioural findings are in contrast to earlier studies on fine motor skills reporting gains (Doyon *et al*., 2009; Huber *et al*., 2004) or at least stabilization (Albouy *et al*., 2013a; Debas *et al*., 2010; Krakauer *et al*., 2005; Krakauer 2009) of motor adaptation performance after sleep but also after diurnal wakefulness (Debas *et al*., 2010) or sleep deprivation (Donchin *et al*., 2002), suggesting that time *per se* rather than sleep is important to engage the consolidation process in motor adaptation.

From a subjective perspective (measured by questionnaires), negative influences of sleep inertia can be excluded (cf. results of the ancovas). However, we are aware that objective measures like performance in a reaction time task (e.g. psychomotor vigilance task) or EEG alpha/theta ratios during a resting condition are lacking.

Regarding sleep measures, we found that subjects spent longer times in N2 sleep after gross motor learning during experimental nap in comparison to a baseline nap without prior learning. This is in line with earlier findings by Peters *et al*. (2007), who found that participants spent significantly more time in stage N2 sleep during an acquisition night (after performing a pursuit rotor task) than during a baseline night. It has to be noted that we did not record a separate acclimatization nap, and therefore ‘first-night’ effects during the baseline nap cannot be excluded. Apart from diurnal nap, the effects of an entire night of sleep (containing multiple sleep phases) on that kind of gross motor consolidation need to be examined carefully. Furthermore, it is well known (Boutin *et al*., 2012) that the best condition for long-lasting retention of motor memory is a multi-session training approach, where practice and testing sessions are alternated. Therefore, a longitudinal study protocol (starting already with recording of the night after the first familiarization phase) should be considered to investigate the impact of sleep on the time course of motor memory consolidation in more detail.

Results presented in this paper indicate that particularly diurnal sleep spindles and REM sleep after gross motor learning seem to counteract a successful consolidation of the new gross motor skill over nap. Higher sleep SpA as well as longer REM sleep durations were found to be related to an over-nap decrease in gross motor performance on the newly learned task by means of reduced steering accuracy. Sleep spindles are proposed as a neurophysiological marker of synaptic potentiation, representing plasticity-related changes in the cortico-striatal motor system following motor learning (Smith *et al*., 2004). However, according to a model by Saletin *et al*. (2011), the role of sleep SpA is important for both strengthening but also targeted forgetting of human memories. Therefore, one could hypothesize that midday sleep may either consolidate or erase new memory contents according to their real-life relevance. As learning to ride an inverse steering bicycle is highly interfering with the everyday needed skill to ride a normal steering bicycle, diurnal sleep spindles might serve as a guardian of ecologically valid memories. Similar to this aspect, Wilhelm *et al*. (2011) found that sleep selectively enhances memory, related to the relevance of the learned material: exclusively those memories that are specifically linked with future expectations are strengthened during sleep. Our data would therefore support the main conclusion that the brain during sleep only consolidates information for future relevance, being definitely not the case for inverse steering bicycling. Fischer *et al*. (2011) recently investigated whether sleep might benefit directed forgetting (stop retrieval of a prepotent memory). They found that especially REM sleep appears to counteract the inhibitory control over prepotent memories by making them even more accessible to retrieval. Facing the dominant inhibition component during riding an inverse steering bicycle, diurnal REM sleep might have similar effects for adapting the highly prepotent process of riding a normal bicycle. With respect to the significance of diurnal sleep, Morita *et al*. (2012) found improved performance in a complex motor skill learning task (three ball cascade juggling) after sleep in comparison to wakefulness. They observed an increase in slow oscillation, delta and sigma EEG spectral power during N3 after juggling. As these changes in EEG spectral power are well known to be critical for the consolidation of explicit knowledge, the authors discussed that even implicit tasks include initially the usage of explicit memory systems, and that in particular complex motor skill learning like juggling requires more time to automatize processes and thus may need a more extensive explicit process. Studies investigating sleep-related cerebral changes mediating memory consolidation of motor skills (Albouy *et al*., 2013b) revealed that the consolidation of new motor sequences that are known explicitly before practice begins seems to require a functional interaction between the basal ganglia (striatum) and limbic (hippocampus) system during post-training sleep. On the other hand, motor skills without explicit knowledge are related to a distinct neural network involving the cerebellum and associated cortical regions (e.g. posterior parietal region, premotor cortex) that revealed to be mostly independent of sleep (Doyon *et al*., 2009). In this vein, Robertson's Awareness Theory (Robertson *et al*., 2004) hypothesizes that sleep benefits off-line gains only when subjects have full explicit knowledge about the motor skill they have to learn. Given that our innovative gross motor task is considered to be mainly an implicit adaptation task, there might be a rather small impact of sleep especially during the early phase of learning. Riding a bicycle whether with normal or inverse steering is constrained by very short time periods that are available for correct steering adjustments (otherwise the rider is enforced to dismount from the bike). The high time pressure on information processing, in turn, mainly hinders the implementation of explicit learning strategies. In contrast to inverted sensory inputs such as in mirror tracing tasks, the motor output solving the original task is inverted through the applied steering apparatus that makes the bicycle initially unridable. During early learning stages, it is supposed to require the inhibition of highly automated movement patterns of normal bicycle riding. Thereby it is quite likely possible that the interference of the old (riding a normal bicycle) on the consolidation of the new gross motor pattern (inverse steering bicycling) is even leading to a performance decrease on the short term. Altogether, this specific kind of adaptation might be influenced by sleep processes not at the novel, but during a later stage, where the new motor skill becomes more automated and inhibition is less prominent. We assume that further studies investigating the impact of sleep on learning a new gross motor skill by adaptation of a highly automated skill have to implement protocols on a long term to control changes in sleep and brain activity until the newly acquired gross motor task approaches asymptotic performance. In sum, our results strengthen the knowledge that motor memory consolidation processes may differ depending on the specific task characteristics, for example importance for everyday life, complexity, number and kind of muscles involved, influence of the environment, duration of the movement execution (utilization of perceptual feedback for movement control or correction), time pressure (utilization of explicit strategies), transfer of past motor experience (new versus adaptation), continuity (discrete, serial or continuous) and pace (self- versus externally paced). This dissociation definitely has to be considered when investigating the effect of sleep on motor memory consolidation.

## SUPPORTING INFORMATION

**Table S1.** Subjective ratings of sleepiness, affectivity, arousal, mood, drive and participation (MDBF, SSS, ASES) before and after retention interval.

**Video S1.** Gross motor task: subjects had to learn to ride a bicycle with inverse steering. The video starts (00:00–00:17) with a demonstration of the utilized bicycle and shows how to handle the inverse steering device. Further it gives an example for a training (00:18–01:55) and a straight-line testing session (01:56–02:11). Finally (02:12–02:46) the assessment of the steering accuracy by means of measuring the steering angle with a rotatory potentiometer is presented.
