# Supplementary material for: The impact of diurnal sleep on the consolidation of a complex gross motor adaptation task
Source: J Sleep Res. 2014 Sep 25;24(1):100–9. doi: 10.1111/jsr.12207 (PMC4491357; doi:10.1111/jsr.12207)
Supplement: Supplementary file 1 — Table S1. Subjective ratings of sleepiness, affectivity, arousal, mood, drive and participation (MDBF, SSS, ASES) before and after retention interval. [file jsr0024-0100-sd1.docx]

**Supplemental Table S1. Subjective ratings of sleepiness, affectivity, arousal, mood, drive and participation (MDBF, SSS, ASES) before and after retention interval.** Data are presented as mean ± SD. Independent-samples t tests (grey) depict the differences in subjective ratings between NAP and NO-NAP. Note: High scores at the ASES_sleepiness and the SSS_sleepiness scale indicate sleepiness, whereas the opposite is true for the scale MDBF_sleepiness, where lower scores represent higher fatigue. Regarding MDBF_affectivity and MDBF_arousal, high scores indicate positive affect and low arousal level, respectively; high scores at ASES_mood and ASES_drive represent happiness and activity, respectively; high scores at ASES_participation indicate apathy. p-values printed in bold represent statistical trends (p<0.10); * highlights p<0.05.

|  | **PRE RETENTION INTERVAL** | | | | **POST RETENTION INTERVAL** | | | |
| --- | --- | --- | --- | --- | --- | --- | --- | --- |
|  | **NAP (n=10)** | **NO-NAP (n=10)** |  | | **NAP (n=10)** | **NO-NAP (n=10)** |  | |
|  | **mean ± SD** | **mean ± SD** | **t(18)** | **p** | **mean ± SD** | **mean ± SD** | **t(18)** | **p** |
| MDBF_sleepiness | 14.20 ± 2.74 | 12.10 ± 3.41 | 1.517 | 0.147 | **13.20 ± 4.39** | **9.90 ± 3.87** | **1.782** | **0.092** |
| MDBF_affectivity | 18.00 ± 1.63 | 16.90 ± 2.73 | 1.095 | 0.288 | 16.00 ± 3.30 | 15.80 ± 3.19 | 0.138 | 0.892 |
| MDBF_arousal | 17.60 ± 1.84 | 14.60 ± 2.46 | **3.091** | **0.006*** | 16.00 ± 2.49 | 14.60 ± 3.03 | 1.129 | 0.274 |
| ASES_sleepiness | 40.20 ± 22.82 | 41.67 ± 30.04 | -0.121 | 0.905 | 37.20 ± 29.60 | 57.80 ± 25.86 | -1.657 | 0.115 |
| ASES_mood | 82.80 ± 12.84 | 79.33 ± 13.17 | 0.581 | 0.569 | 73.30 ± 16.75 | 65.70 ± 20.70 | 0.902 | 0.379 |
| ASES_drive | 32.50 ± 24.71 | 31.78 ± 23.38 | 0.065 | 0.949 | 42.80 ± 30.57 | 57.30 ± 26.01 | -1.142 | 0.268 |
| ASES_participation | 79.70 ± 13.74 | 69.00 ± 16.08 | 1.565 | 0.136 | **71.80 ± 19.14** | **55.10 ± 18.11** | **2.004** | **0.060** |
| SSS_sleepiness | 2.40 ± 0.97 | 2.78 ± 1.30 | -0.723 | 0.479 | **2.70 ± 1.16** | **3.90 ± 1.10** | **-2.374** | **0.029*** |
