# Supplementary material for: The impact of diurnal sleep on the consolidation of a complex gross motor adaptation task
Source: J Sleep Res. 2014 Sep 25;24(1):100–9. doi: 10.1111/jsr.12207 (PMC4491357; doi:10.1111/jsr.12207)
Supplement: Supplementary file 3 — Video S1. Gross motor task: subjects had to learn to ride a bicycle with inverse steering. The video starts (00:00–00:17) with a demonstration of the utilized bicycle and shows how to handle the inverse steering device. Further it gives an example for a training (00:18–01:55) and a straight-line testing session (01:56–02:11). Finally (02:12–02:46) the assessment of the steering accuracy by means of measuring the steering angle with a rotatory potentiometer is presented. [file jsr0024-0100-sd3.docx]

**Supplemental Video. Gross motor task: subjects had to learn to ride a bicycle with inverse steering.** The video starts (00:00-00:17) with a demonstration of the utilized bicycle and shows how to handle the inverse steering device. Further it gives an example for a training (00:18 – 01:55) and a straight-line testing session (01:56-02:11). Finally (02:12-02:46) the assessment of the steering accuracy by means of measuring the steering angle with a rotatory potentiometer is presented.
